# Microbial Community Dynamics and Metabolite Changes during Wheat Starch Slurry Fermentation

**DOI:** 10.3390/foods13162586

**Published:** 2024-08-18

**Authors:** Xiaoping Li, Yujin Yang, Xin Fan, Xinzhong Hu

**Affiliations:** College of Food Engineering and Nutritional Science, Shaanxi Normal University, Xi’an 710062, China; yujinyyyy@163.com (Y.Y.); 15091478392@163.com (X.F.); hxinzhong@snnu.edu.cn (X.H.)

**Keywords:** natural fermentation, wheat starch, microbial dynamics, HS-SPME/GC-MS

## Abstract

Wheat starch fermentation slurry is the main substrate for producing Ganmianpi, a traditional Chinese fermented wheat starch-based noodle. In the present work, the microbial population dynamics and metabolite changes in wheat starch fermentation slurry at different fermentation times (0, 1, 2, 3, and 4 days) were measured by using high-throughput sequencing analysis and headspace solid-phase microextraction gas chromatography–mass spectrometry (HS-SPME/GC-MS) methods. The texture and sensory properties of Ganmianpi made from fermented starch slurry are also evaluated. The results showed that *Latilactobacillus curvatus* and *Leuconostoc citreum* were the dominant bacteria in wheat starch fermentation slurry, while *Saccharomyces cerevisiae* and *Kazachstania wufongensis* were identified as the main species of fungi. With the extension of fermentation time, the reducing sugar content first increased and then decreased, when the titratable acidity content showed an increasing trend, and the nonvolatile acid was significantly higher than the volatile acid. A total of 62 volatile flavor compounds were identified, and the highest content is alcohols, followed by acids. Fermentation significantly reduced the hardness and chewiness of Ganmianpi, and increased its resilience and cohesiveness. Ganmianpi made from fermented starch slurry for two and three days showed a higher sensory score than other samples. The present study is expected to provide a theoretical basis for exploiting the strains with potential for commercial application as starter cultures and quality improvement of Ganmianpi.

## 1. Introduction

**Figure 1:**
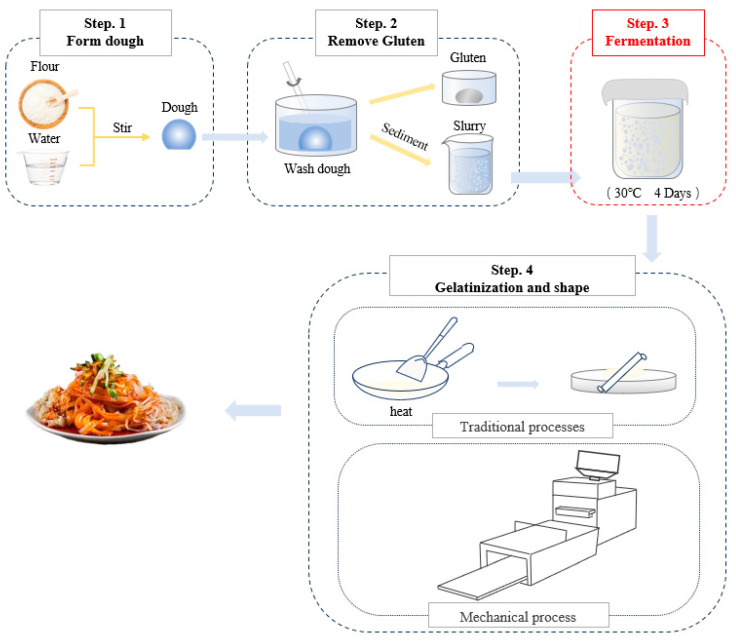
The main production process of Ganmianpi.

Ganmianpi is a kind of Liangpi, which is a traditional Chinese wheat starch gel food and is commonly consumed in northwest China, especially in Shaanxi [1,2]. Liangpi is very popular because of its unique flavor, taste, and texture, and has a wide market in China [1,2]. Unlike Liangpi, Ganmianpi is usually made from fermented wheat starch through four processes (Figure 1). First, adding water to the wheat flour to knead into dough. Then, the dough was washed in water to remove gluten and obtain the starch slurry. Next, the starch slurry is fermented. Finally, the starch slurry is ripened and compressed or rolled into sheeting, and cut into strips for consumption.

Fermentation is a crucial step in the production of Ganmianpi, and it is also a unique step different from Liangpi. Fermentation can increase acidity, decline pH, and prolong the shelf life of food [3], which is beneficial for Ganmianpi and also a critical issue to be solved for Ganmianpi [3]. Meanwhile, fermentation makes fermented cereal-based products generally more enjoyable and digestible, with good nutritional features, and desired texture [4]. In the previous study, we found that fermentation significantly modified the multi-scale structure and physicochemical properties of wheat starch [5], and sodium alginate and konjac gum showed a beneficial impact on improving the quality of fermented wheat starch-based foods [6]. At present, the fermentation of starch slurry for Ganmianpi is mainly based on the spontaneous fermentation of microorganisms from raw materials and the environment. Spontaneous fermentation is cheap and ready to implement, and the autochthonous microbiota colonized in the raw materials and indigenous environment could give the production with the regional flavor characteristics. However, due to the diversity and complexity of indigenous microorganisms, there are challenges in ensuring consistency in the flavor and quality of final products across different batches [7]. Screening dominant fermentation bacterial species, and making and employing starter is a solution to ensure the quality and safety of fermentation products. Therefore, it is fundamental and necessary to investigate the succession of microbial community structure and changes in metabolic products during the fermentation process in the wheat starch slurry for Ganmianpi production. However, to our knowledge, there has been no study on the microbial population dynamics and metabolite changes during wheat starch slurry fermentation for Ganmianpi.

In recent decades, the combination of high-throughput sequencing technology and HS-SPME-GC-MS has been widely applied in the study of microbial community structure and characteristic flavor substances in various fermented foods, such as dark teas [8], jujube wine [9], and Chinese sauerkraut [10]. Therefore, this study aims to clarify the dominant bacterial and fungal species by exploring the changes and trends in microbial community structure and diversity during the fermentation of wheat starch slurry through high-throughput sequencing technology. At the same time, the present work was expected to be able to elucidate the types of the characteristic flavor substances in fermented wheat starch slurry and provide the theoretical basis for stabilizing and improving the taste, flavor, and quality of Ganmianpi.

## 2. Materials and Methods

### 2.1. Materials

Wheat flour was purchased from Xi’an Aiju Grain and Oil Industrial Group. (Xi’an, China). Wheat starch was purchased from Henan Zhengzhou Midan’er Food Co., Ltd. (Zhengzhou, China). Lysozyme and protease K are all purchased from Shanghai Shenggong Co., Ltd. (Shanghai, China). PowerSoil^®^ DNA Isolation Kit, agarose gel electrophoresis-related reagents and PCR amplification kits were purchased from Takara Biomedical Technology (Beijing) Co., Ltd. (Beijing, China).

### 2.2. Preparation of Fermented Wheat Starch Slurry

Wheat flour was mixed with water in a ratio of 2:1 (*w*/*w*), stirred, and mixed to form the dough, and then proofed for 30 min. Water was added to the dough, constantly rubbed and washed. Repeat the steps above approximately five times to remove insoluble gluten to obtain starch slurry. Then, the wheat starch was added to the slurry so that the starch slurry was adjusted to a concentration of 22° Bé. The slurry was placed at 30 °C for fermentation 96 h. Five samples from different fermentation times were collected and marked for F0 (0d), F1 (1d), F2 (2d), F3 (3d), and F4 (4d). Some samples were stored at −80 °C for DNA extraction and microbial analysis, the other samples were immediately measured for pH, reducing sugars, and volatile flavor compounds.

### 2.3. Microbial Analysis of Fermented Starch Slurry

#### 2.3.1. DNA Extraction and PCR Amplification

According to previous literature [11], total DNA was extracted using MagPure Soil DNA LQ Kit (Takara Biomedical Technology (Beijing) Co., Ltd. (Beijing, China). The 16S rRNA V3 + V4 region and ITS1 region were amplified for bacteria and fungi, respectively. Retrieve and elute based on the preliminary quantitative results of electrophoresis. Quantitative detection was performed using the Quant-iT PicoGreen dsDNA Assay Kit system from Thermo Fisher Scientific Inc. (Shanghai, China). Purified amplicons were paired-end sequenced on an Illumina MiSeq platform (Illumina, San Diego, CA, USA) according to standard protocols.

#### 2.3.2. Illumina MiSeq Sequencing and Processing of Sequencing Data

Raw FASTQ files were demultiplexed, and operational taxonomic units (OTUs) were clustered with a 97% similarity cutoff using Usearch. By using QIIME2 (2020.6) software, default parameters were used, and a naive Bayesian classifier was used to annotate the feature sequence of each OTU using UNITE as the reference database. We compared the bacterial 16S rRNA gene dataset with the Silva 138 dataset, and compared the fungal ITS gene data set with the nt database. We obtained OTU analysis table and alpha/beta diversity by using a Python script for QIIME2.

### 2.4. Analysis of Physicochemical Characteristics of Starch Slurry and Ganmianpi

The content of reducing sugar was detected by the 2,3-dihydroxy succinate method. The pH of the slurry was measured with a pH meter (OHAUS, STARTER 2C). Titratable acidity (TTA) was determined by titration with 0.1 M NaOH until neutral.

### 2.5. Analysis of Volatile Flavor Compounds in Fermented Starch Slurry

The flavor compounds in the samples were analyzed by HS-SPME/GC-MS, according to the procedure described by Liu et al. [12]. The volatile compounds were extracted by an automatic headspace sampling system (CTC HTS PAL, Basel, Switzerland) with a 50/30 μm fiber (DVB/CAR/PDMS). Each starch slurry sample (5 mL) and internal standard 2-octanol was placed in a 15 mL SPME glass vial and stirred. After extraction, the fiber was desorbed at 250 °C for 5 min. Volatile flavor compounds were separated in an Agilent 5975B-GCMS (NYSE: A). The column was HP-3 MS column: (30 m × 0.25 mm i.d., 0.25 µm film thickness). The carrier gas helium with a flow rate of 1 mL/min. The oven temperature was held at 40 °C for 3 min, raised to 150 °C at a rate of 3 °C/min and holding for 2 min, and raised to 250 °C at a rate of 5 °C/min. The ion source and transfer line temperatures were set at 230 and 250 °C, respectively. The ion energy for electron impact (EI) was always 70 eV.

### 2.6. Preparation of Ganmianpi

The fermented wheat starch slurry from different days was used as raw material for preparing Ganmianpi, and the WCJX-45 mechanism (weichuang, Shenzhen, China) was used to make the sample. Starch slurry is added to the hopper and is extruded and matured by a single screw (at a temperature of approximately 85 °C). The matured starch gel is rolled into sheeting (with a thickness of approximately 0.6 mm), that is Ganmianpi. Ganmianpi made from the fermentation solution F0 (indicating the unfermented wheat starch slurry) is labeled G0, and the other Ganmianpi samples prepared with fermented starch slurry for 1 d, 2 d, 3 d, 4 d are labeled G1, G2, G3, and G4, respectively.

### 2.7. Texture Profile Analysis of Ganmianpi (TPA)

The texture properties of samples were tested by TA.XT. Plus texture analyzer (Stable microsystem, Godalming, UK). For every texture test, four layers of samples were prepared and stacked together, maintaining the size of 5 × 5 × 4 mm. The samples were put on the measurement platform of the physical property tester for TPA and analyzed by the P/50 test probe. This experiment’s test mode and parameters were set as follows: pre-test speed, test speed, and post-test speed are all 1 mm/s, the stress variable is 50%, and the trigger force is 5 g, respectively.

### 2.8. Sensory Evaluation Method of Ganmianpi

In this experiment, a total of 11 trained panelists (6 female, 5 male) participated in sensory evaluation. Participants were familiar with Ganmianpi taste and had previous experience in sensory evaluation. Samples were numbered and placed randomly and scored according to the scoring criteria in Supplementary Table S1, including color, surface smoothness, elasticity, toughness, chewiness, and flavor. The protocol and procedures employed were ethically reviewed and approved prior to the start of this study. The sensory evaluation standards of Ganmianpi are listed in Supplementary Table S1.

### 2.9. Statistical Analysis

One-way analysis of variance (ANOVA) analysis of SPSS 21.0 (SPSS Inc., Chicago, IL, USA) was used for statistically significant differences in raw data. Correlations between bacterial communities were analyzed using the Mantel procedure in R-3.6.x software.

## 3. Results and Discussion

### 3.1. Changes in Microbial Communities

The bacterial and fungal communities’ rarefaction curves based on OTUs with a 97% similarity are shown in Supplementary Figure S1A,B. The OTU increased rapidly with the increase in sequencing depth. After exceeding 10,000 sequences, the OTU of bacteria and fungi increased slowly and reached the saturation plateau. Supplementary Figure S1C,D also show that the Shannon diversity index curves of bacteria and fungi in the slurry have flattened. These results indicate that the current sequencing depth is sufficient to reflect the true microbial diversity of wheat starch slurry solution [13].

#### 3.1.1. Analysis of Bacterial Community Dynamics during Fermentation

**Figure 2:**
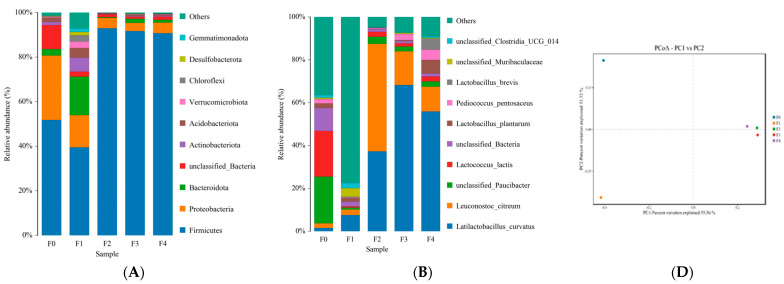

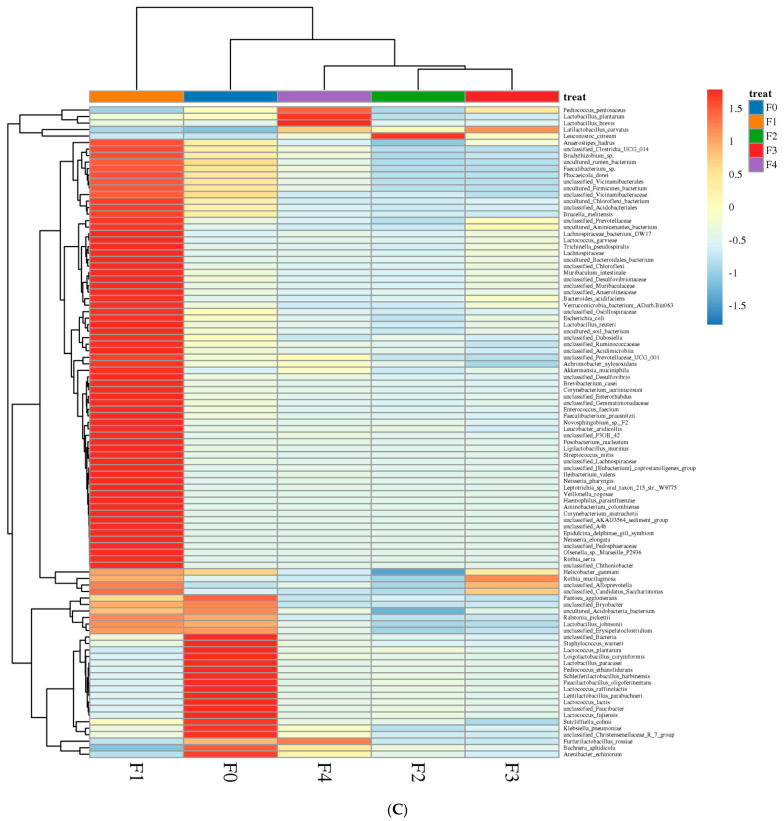
Changes in the bacterial community and diversity of samples. The relative bacterial abundances are at the level of phylum (**A**) and the species (**B**), respectively. Heatmap showing the phylogenetic distribution of the bacterial samples (**C**). Principal coordinate analysis of each sample colored according to the fermentation stages in bacteria (**D**). Five samples from different fermentation times were marked for F0 (0d), F1 (1d), F2 (2d), F3 (3d), and F4 (4d).

The analysis of bacterial community dynamics during fermentation is shown in Figure 2. For bacteria at the phylum level (Figure 2A), *Firmicutes* and *Proteobacteria* were predominant in the original and early fermentation samples (F0 and F1 in Figure 2A). With the extension of fermentation, the bacterial richness decreased significantly. It may be because of their intolerance of anaerobic and high-acid fermentation environments. Comparing the microbial colony structure of different fermentation days of starch slurry, the community structure of F1 was relatively complex. In addition to *Firmicutes* and *Proteobacteria, Bacteroidota* and *Actinobacteria* also show a relatively high abundance. These changes may be because of the rich nutrients contained in wheat starch slurry, and conditions (neutral, 30 °C) are suitable for the growth and reproduction of microorganisms carried in starch and derived from the environment. With the prolonged fermentation time, the dynamic changes in the bacterial community gradually became consistent, and *Firmicutes* became the main bacteria at fermentation for 2 d, 3 d, and 4 d, with abundances of 92.97%, 91.77%, and 90.75%, respectively.

Figure 2B shows bacterial community dynamics at the species level. It can be seen that the diversities of bacterial communities in the five samples were similar, but their abundances show significant differences. *Lactococcus lactis* in F0 exhibits higher abundance than other samples, and its relative abundance reduced when *Latilactobacillus curvatus* and some unidentified bacteria increased in F1. Similar to the phylum level, the bacterial communities of F0 and F1 at the species level were complex. Many bacteria were not identified in the F1 sample at the species level, possibly due to complex species. This could be attributed to the appropriate fermentation condition for the growth and reproduction of various bacteria carried in wheat starch and derived from the environment, as described at the phylum level. *Latilactobacillus curvatus* and *Leuconostoc citreum* can be seen to exist in all five samples, and they became the leading genera in the starch slurry after fermentation for 2 d, accounting for 87% of the relative abundance of the total bacteria, making them the most important bacteria in this fermented starch slurry. *Latilactobacillus curvatus* (*synonym Lactobacillus curvatus*) [14] has gained widespread attention due to its excellent fermentation properties and health benefits [15]. It is considered ubiquitous in the foods with lactic acid fermentation originated from the environment [16]. *L. curvatus* showed versatile carbohydrate metabolism, acidic tolerance, a good growth rate, and a good acidification rate in the rye sourdoughs; simultaneously, it is capable of improving the quality of bread and reduce acrylamide content in mixed rye—wheat bread as starter [17]. In addition, a previous study also confirmed that *L. curvatus* could be a predominant species in a kind of French organic sourdough [18]. *Leuconostoc citreum* exists in various types of fermentation products. It can utilize glucose to produce lactic acid, acetic acid, alcohol, mannitol, CO_2,_ and aromatic compounds [19], which make essential contributions to the special flavor of fermented foods [19,20]. Its abundance was reduced in samples with fermentation for 3 d and 4 d, but it is still the second leading lactic acid bacteria besides *L. curvatus*. With a prolonged fermentation time, the content of *L. curvatus* in the slurry increased, and the quantities of other bacteria markedly decreased. The high acid conditions and the increasing number of *L. curvatus* intensified the competition, which is not conducive to the growth of other bacteria.

Figure 2C shows the heat map of the bacterial community at the species level. It can be seen that there was a low amount of bacteria species in F2, F3, and F4, whereas F0 and F1 have a high number of bacteria species and diversity. The bacteria species and diversity of F0 were lower than that of F1, and higher than that of F2, F3, and F4, and were markedly distinct from other samples because of bacteria that originated from the raw material at the initial fermentation. Sample F1 exhibited the highest bacterial abundance and diversity, which is due to that various bacteria grew and reproduced rapidly in suitable nutrition and environment. However, after one day of fermentation, the *Latilactobacillus curvatus* and *Leuconostoc citreum* rapidly dominated the bacterial community. The result is consistent with the relative abundance analysis, as shown in Figure 2B.

The principal coordinate analysis (PCoA) result of bacteria is shown in Figure 2D It can be found that five samples were separated into three clusters, demonstrating differences between the bacterial communities of these samples. F1 was located in the upper left part, and F0 and F1 were far apart and had significant differences, forming independent groups. F2, F3, and F4 flocked together in the PCoA map, indicating there were no significant differences between bacterial communities on F2, F3, and F4, illustrating that the three samples had similar bacterial community structures and that the bacterial community structure was stable at the third fermentation day. This result correlates with the heatmap shown in Figure 2C. With prolonged fermentation, *Latilactobacillus curvatus* and *Leuconostoc citreum* became the leading bacteria, which maybe the main reason for the differences. This result also suggested that the growth and reproduction of *L. curvatus* and *L. citreum* during fermentation inhibited other bacteria. The present result supported previous discoveries that fermentation treatments could cause the beneficial bacteria to grow rapidly and inhibit the growth of undesirable bacteria [21].

It can be seen from the changes in the bacterial community that the natural fermentation process of the starch slurry was a mixed fermentation of various bacteria. The main bacteria were *Latilactobacillus curvatus* and *Leuconostoc citreum*. On the third day of fermentation (F3), *Latilactobacillus curvatus* and *Leuconostoc citreum* became the dominant bacteria at the species level. The proportion of *Latilactobacillus curvatus* and *Leuconostoc citreum* is 68.28% and 15.72%, respectively, a total of 84.00%. The highest relative abundances of *Latilactobacillus curvatus* in wheat starch fermentation imply its potential as a fermenting agent for Ganmianpi.

#### 3.1.2. Analysis of Fungal Community Dynamics during Fermentation

**Figure 3:**
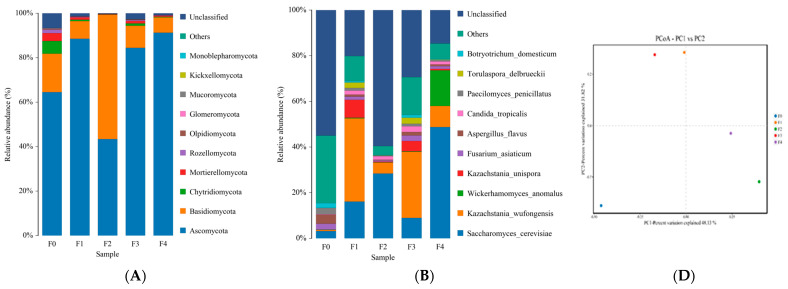

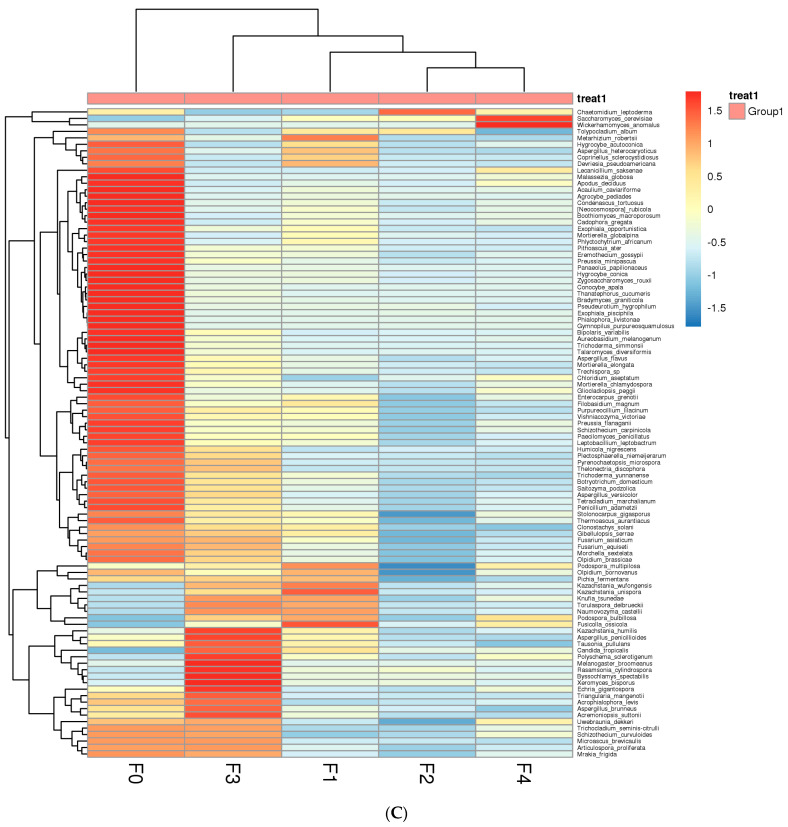
Changes in the fungi community and diversity of samples. The relative fungi abundances at the level of phylum (**A**) and the species (**B**), respectively. Heatmap showing the phylogenetic distribution of the fungi samples (**C**). Principal coordinate analysis of each sample colored according to the fermentation stages in fungi (**D**). Five samples from different fermentation times were marked for F0 (0d), F1 (1d), F2 (2d), F3 (3d), and F4 (4d).

Figure 3A,B represent the microbial community structure of fungi at the phylum and species levels, respectively. At the fungal phylum level (Figure 3A), *Ascomycota* and *Basidiomycota* were the main fungi, taking a large proportion of microbial community structure. *Ascomycota* was the dominant phylum in the other four samples except sample F2. *Basidiomycota* had a more significant proportion in F2. After two days of fermentation, the fungal community structure became stable and *Ascomycota* dominated with an abundance of 84.47% in F3 and 91.28% in F4. The present result was consistent with the report on the fungal community of fresh fermented rice noodles at the phyla level [22]. *Ascomycota* is one the most diverse and universal phylum of eukaryotes and also the largest phylum of fungi [23].

At the level of fungal species (Figure 3B), it can be seen that species in F0 are complicated and diverse, which may have originated from wheat flour and starch materials and the fermentation environment. With the extension of fermentation, *Saccharomyces cerevisiae* and *Kazachstania wufongensis* accounted for a large proportion. The proportion of *S. cerevisiae* was the highest in F2 and F4, while *K. wufongensis* had the highest proportion in F1 and F3. The differences in dominant fungi may be caused by differences in fermentation duration [24]. Microbial community dynamics in sourdough [25,26] and natural sour starters [27] arouse the interest of researchers to enhance the quality of traditional food. In a recent study, Wang et al. [25] reported that *Saccharomyces cerevisiae* and *Kazachstania humilis* were the dominant fungal species of type Ι sourdough steamed bread, and the latter was the most predominant. The dominant fungal genera in whole-wheat traditional sour starter were *Saccharomyces. cerevisiae, Candida glabrata,* and *Zygosaccharomyces* spp. [27]. *Kazachstania wufongensis* is a new yeast species found from soil by Lee et al. [27], who indicated that it and *Kazachstania exigua* are closely related phylogenetically. *Kazachstania exigua*, *Saccharomyces cerevisiae*, *Torulaspora delbrueckii* and *Wickerhamomyces anomalus* were found to be typical yeast present in stable sourdough in an early literature [26]. The latter three have also been identified in the current starch slurry. The fungal composition of fresh fermented natural rice noodles was varied and complex, including *Curvularia* and *Aspergillus*, which were identified as human pathogens, indicating the spontaneously fermented was not guaranteed the safety of fresh fermented rice noodles [22]. However, *Saccharomyces cerevisiae* was selected for the strain of fresh fermented rice noodles, *S. cerevisiae* dominated at the 0 h stage and increased significantly, and accordingly restrained the growth of other fungi. The present result indicated that *S. cerevisiae* is the most important fungus in the completed fermentation wheat starch slurry (F4), and its role and application in fermented food implies its potential as a fermenting agent fungal for Ganmianpi.

The fungal community heatmap (Figure 3C) showed that F0 contained the highest number of fungal species, which may be due to fungal diversity originating from the raw material and the surrounding environment during the initial fermentation. With prolonged fermentation, the number and abundance of fungi decreased. The increase in abundance in dominant fungi (*Saccharomyces cerevisiae* and *Kazachstania wufongensis*) is the main reason for the decline in diversity. Samples F2 and F4 had similar diversity, which may be because F2 and F4 showed a low amount of other fungi, besides the finding that F2 contained high *Chaetomidium leptonema*, whereas *Saccharomyces cerevisiae* and *Kazachstania wufongensis* were the dominant fungi of F4. However, a larger variation was observed in F1 and F3. The result is consistent with the relative abundance report as shown in Figure 3B. The present result indicated that the fungal diversity in wheat flour and starch material was diverse, and the fermentation environment was varied and uncontrollable, thus strongly influencing the fungal community structures during the fermentation of wheat starch slurry. A similar finding has been reported in fresh fermented rice noodles by Wang et al. [22].

The result of fungal PCoA, as shown in Figure 3D, exhibited that the distance between five samples with different fermentation days was considerable, indicating significant differences in the dynamic changes in fungal communities during fermentation. However, it can be seen that F1 and F3 were located in the second quadrant and had a closer distance when F2 and F4 dispersed in the fourth quadrant. F0 is situated in the lower left corner of the first quadrant and far away from the four fermentation samples. The result correlates with the heatmap shown in Figure 3B. The increase in fungal community composition differences may be due to differences in abundance among *Saccharomyces cerevisiae*, *Kazachstania wufongensis* and other fungi, which originated from varied and uncontrollable fungi of the fermentation environment, as described in the heatmap section.

It can be seen from the changes in the microbial community that the natural fermentation of wheat starch slurry for the Ganmianpi product was a mixed fermentation of various microorganisms. The main bacteria were *Latilactobacillus curvatus* and *Leuconostoc citreum*. The main fungi were yeast (mainly including *Saccharomyces cerevisiae* and *Kazachstania wufongensis*). In particular, considering their unique role and application in other fermentation food, *Latilactobacillus curvatus* and *Saccharomyces cerevisiae* show their potential as a mixed bacterial fermentation agent for Ganmianpi products.

### 3.2. Changes in Organic Acids and Reducing Sugars

**Figure 4:**
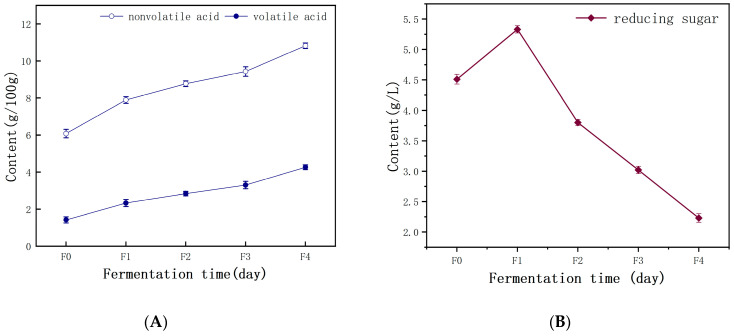
Physicochemical properties of wheat starch slurry during fermentation. (**A**) Changes in organic acid contents; (**B**) changes in reducing sugar. Five samples from different fermentation times were marked for F0 (0d), F1 (1d), F2 (2d), F3 (3d), and F4 (4d).

Organic acids, including volatile acids (mainly acetic acid) and nonvolatile acids (mainly lactic acid), are essential taste components in cereal fermentation food [28]. The change in organic acids during the fermentation of starch slurry is shown in Figure 4A. The content of volatile and nonvolatile acids gradually increased during fermentation. The increase in organic matter may be due to the growth and metabolic activity of microorganisms. Lactic acid bacteria proliferated in large quantities, and converted starch and glucose into nonvolatile acids such as lactic acid. Research has found that regulating acidification significantly impacts fermentable carbohydrates, which affects bacterial cell production and the production of lactic acid and acetic acid by affecting carbohydrate metabolism [29].

Reducing sugar is not only a sweet substance but also an energy source for microorganisms during fermentation. The content of carbohydrates in starch slurry is an important parameter, which was generally used as a carbon source by bacteria for fermentation. During fermentation, the main forms of reducing sugars were maltose and glucose, providing a carbon source for microbial metabolic activities [4]. As presented in Figure 4B, the reducing sugar content gradually increased in the early stages and subsequently decreased later. The increase in reducing sugar content during the early stage was caused by the decomposition of starch in the raw materials. It may be due to organic acids accumulated during microbial fermentation that can hydrolyze starch. The amylase in wheat flour and some yeast can decompose starch into reducing sugars, which significantly increases reducing sugar content [30]. In the later stage, a large amount of reducing sugar was utilized by microbial metabolic activities, resulting in a decrease in reducing sugar content [8].

### 3.3. Analysis of Volatile Compounds during Fermentation

Volatile flavor compounds endow fermented food with unique flavor, which helps improve the food’s sensory characteristics and is an important indicator. As shown in Table 1, 62 volatile flavor substances were detected in five samples, including 14 alcohols, 15 esters, 11 acids, and 22 other flavor compounds. The substance with the highest content in all flavor substances is alcohol, followed by acids.

Alcohols are secondary products derived from yeast during alcohol fermentation by utilizing amino acids or sugar metabolism [31]. Generally, all alcohol flavoring substances contain ethanol, which produces a pleasant aroma. Ethanol is a vital aroma substance that can be converted into esters and other components. Some yeast can convert ethanol into aldehydes, esters, and other substances [32]. As shown in Table 2, the ethanol content increased and then decreased during fermentation. On the second day of fermentation, phenyl ethyl alcohol appeared, and its content gradually decreased as fermentation progressed. Phenylethyl alcohol has a sweet and elegant aroma as an aromatic higher alcohol [33]. Appropriate acid content can enrich the taste and texture of fermented food. The acetic acid content continuously increased during fermentation, giving the starch slurry a sour taste. The hydrolysis of triglycerides, lipid oxidation, and the conversion of aldehydes or ketones might both obtain acids [34].

Numerous literatures reported that spontaneous fermentation involves a complex microbial community and enzyme-promoting course, which influenced the volatile component dynamics and provides a unique flavor of fermentation food. The increase in alcohol content is related to fungi, especially *Saccharomyces*. Sufficient evidence suggests *Saccharomyces* are important microorganisms in alcohol production and have efficient saccharification ability. At the same time, *Saccharomyces* can produce flavor substances such as esters, alcohols, and organic acids, which mainly play a fragrance role in food [35,36]. The increase in acidity is a result of the activity of lactic acid bacteria. Previous studies have shown that *Latilactobacillus curvatus* and *Leuconostoc citreum* plays a crucial role in increasing acidity during the fermentation process and can promote its flavor and aroma [17,19,37].

On the first day of fermentation (F1), the alcohols and esters in the starch slurry reached their maximum. The types and quantities of flavor compounds increased on the second and third days of fermentation. Ethanol, octanol, phenylethyl alcohol, ethyl octanoate, ethyl decanoate, and other flavor compounds resulted in the product containing a unique aroma. Compared to the second and third day of fermentation, the acidity increased significantly, while alcohols, esters, and total volatile components did not change significantly. However, on the fourth day of fermentation, the acidity continued to increase significantly, but ethanol and total volatile components significantly decreased. This result indicated that appropriate fermentation time (1 d–3 d) is beneficial for the formation of volatile components, but prolonged fermentation may lead to a large accumulation of acid, which can damage the flavor of Ganmianpi.

### 3.4. Ganmianpi Quality Analysis

**Figure 5:**
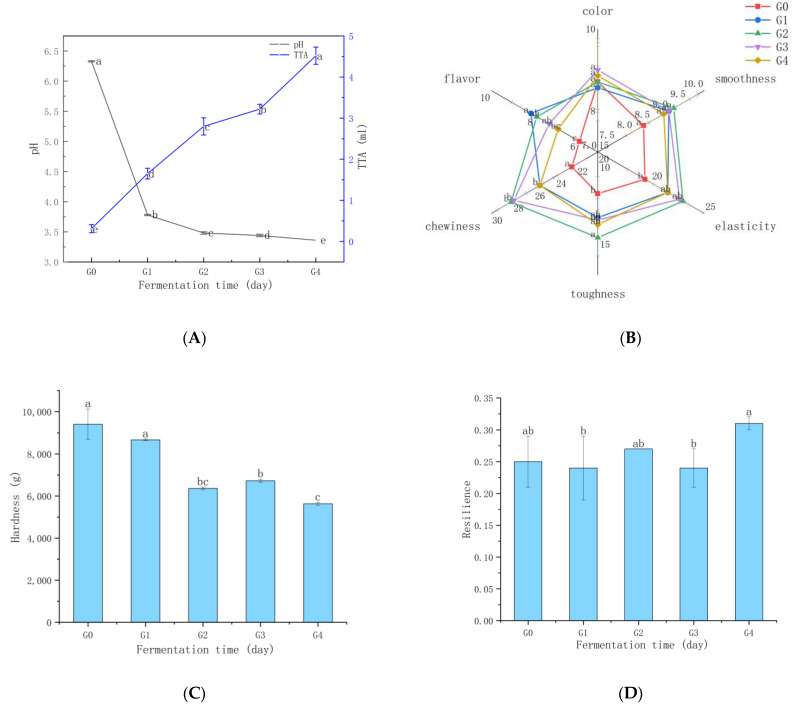

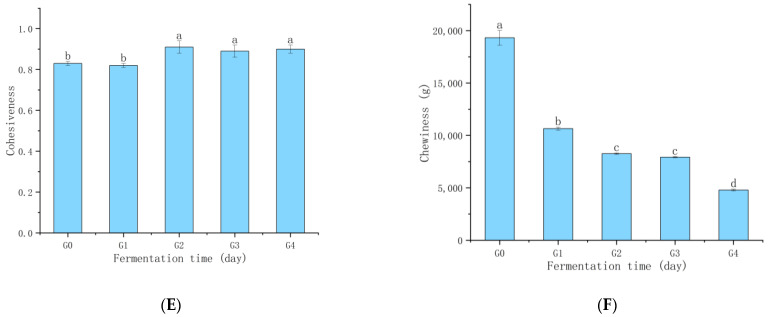
The quality properties of Ganmianpi: (**A**) pH and TTA of samples fermented at different times, (**B**) sensory scores of samples fermented at different times, (**C**–**F**) texture properties of samples: (**C**) hardness, (**D**) resilience, (**E**) cohesiveness, and (**F**) chewiness. Different superscript letters indicate significant differences at *p* < 0.05 (Duncan’s multiple comparisons post hoc tests).

Wheat starch slurry with different fermentation times is made into Ganmianpi. The pH, titratable acidity (TTA), texture, and sensory properties of Ganmianpi were evaluated. Figure 5A shows the changes in pH and TTA of Ganmianpi made from wheat starch slurry with different fermentation times. The pH of Ganmianpi gradually decreased, while TTA increased significantly, which were similar to the change in titratable acidity in the starch fermentation slurry samples. The present result was also consistent with these reports on the change in pH and acidity in cereal fermentation food [28]. The change in acidity has a more significant impact on both the processing and the final quality, such as the texture and sensory properties of Ganmianpi. We found the unfermented wheat starch slurry was challenging to form viscoelastic and smooth structure of Ganmianpi during processing with high viscosity, inelasticity. After only one day of fermentation, Ganmianpi quality was improved, which can be reflected in the change in texture properties of Ganmianpi, as shown in Figure 5C–F. Unfermented sample (G0) has the highest hardness and chewiness. The hardness and chewiness of Ganmianpi showed a significant decrease with the extension of fermentation time, which gave people a soft feeling in sensory evaluation. Resilience showed little change in samples of different fermented stages, and cohesiveness showed an increased trend in the later stages of fermentation. This result is similar to the previous study [38], which may be caused by gas produced from microbial metabolism during fermentation, forming porous structures and showing lower firmness. In addition, in our previous study, we found that fermentation could change the thermal and pasting properties of the wheat starch gel, thus affecting the texture properties of the product [39].

Sensory properties (detailed sensory evaluation standards were shown in Supplementary Table S1) of Ganmianpi made from wheat starch slurry with different fermentation times was evaluated, and the results are shown in Figure 5B. From the results of sensory scores, G2 and G3 had higher scores, which are 91.42 and 89.25, respectively. There was no significant difference between G2 and G3. Both of them present better edible quality than other samples. G0 and G4 have lower scores than G2 and G3, and the score of sample G0 was the lowest, which fully indicated that fermentation was beneficial in improving the sensory quality of Ganmianpi. There were no significant differences in color among the five samples. The smoothness, elasticity, toughness, and chewiness of Ganmianpi made from fermented wheat starch slurry obtained the higher scores than that of un-fermentation. The scores of samples G2 and G3 in toughness, and chewiness were highest. However, with the extension of fermentation time, the scores of toughness, and chewiness in G4 were significantly lower than G2 and G3. The change in sensory scores in smoothness, elasticity, toughness, and chewiness is related to the texture properties, as described in the TPA section, which may be mainly caused by the changes in the multi-scale structure of starch induced by fermentation [5,22]. It is worth noting that the flavor scores of Ganmianpi have undergone significant changes with the extension of fermentation time. The flavor scores of Ganmianpi prepared from fermented starch slurry are higher than those of unfermented samples (G0), which may be due to the production of large amounts of volatile substances during fermentation and the improved flavor of Ganmianpi. As the fermentation time increases, the flavor score of Ganmianpi first increases and then decreases. The samples of G1 and G2 have the highest flavor score, and the decrease in the flavor score in the G4 sample might be due to the accumulation of excessive acid, which is consistent with the changes in volatile substances, as shown in Table 1 and Table 2.

In general, fermentation of wheat starch slurry can improve the texture and sensory properties. Samples with G2 and G3 had higher sensory scores, indicating that the fermentation time was crucial to the quality improvement of Ganmianpi, and appropriate fermentation times of wheat starch slurry were 2 d and 3 d for Ganmianpi processing.

## 4. Conclusions

In this study, we demonstrated that the microbial community structure of starch slurry underwent dynamic evolution during fermentation, and the natural fermentation process of Ganmianpi was a mixed fermentation of various microorganisms. The dominant bacteria were *Latilactobacillus curvatus* and *Leuconostoc citreum* throughout the fermentation period. *Saccharomyces cerevisiae* and *Kazachstania wufongensis* were the leading genera of the fungal species. Considering their unique role and application in fermentation food, *Latilactobacillus curvatus* and *Saccharomyces cerevisiae* show their potential as a mixed bacterial fermentation agent for Ganmianpi products. We found that fermentation significantly influenced the texture properties and sensory score of Ganmianpi. We also elucidated the dynamic changes in flavor substances and physicochemical characteristics during the fermentation process of wheat slurry, which could provide a theoretical basis for stabilizing and improving the taste, flavor, and quality of Ganmianpi.

## Figures and Tables

**Table 1:** Classification of chemical constituents identified.

No.	Compound	Species Number	Relative Content (%)				
			F0	F1	F2	F3	F4
1	alcohols	14	57.16	86.05	86.11	82.32	67.22
2	esters	15	24.59	4.3	2.71	4.32	4.24
3	acids	11	3.02	2.09	4.06	8.66	15.46
4	others	22	2.98	5.31	3.22	0.91	3.06
5	total	62	87.75	97.75	96.10	96.21	89.98

**Table 2:** Metabolites of main volatile compounds in starch slurry during fermentation.

Number	Compound	Relative Content (%)				
		F0	F1	F2	F3	F4
1	Ethyl alcohol	44.44	69.59	69.50	63.95	51.22
2	2-Octanol	8.48	15.26	9.49	11.95	10.89
3	Phenylethyl Alcohol	--	--	6.95	5.59	4.42
4	Octanoic acid, ethyl ester	--	--	0.50	0.58	0.40
5	Hexadecenoic acid, ethyl ester	5.83	1.78	0.63	1.04	1.73
6	Ethyl Oleate	6.46	1.57	--	--	--
7	Acetic acid	--	0.67	1.55	6.12	10.52
8	Octanoic Acid	0.38	0.66	1.07	1.27	1.65
9	n-Decanoic acid	--	--	0.68	0.76	1.38

## Data Availability

The original contributions presented in this study are included in this article and Supplementary Material. Further inquiries can be directed to the corresponding author.

## Supplementary Materials

The following supporting information can be downloaded at: https://www.mdpi.com/article/10.3390/foods13162586/s1, Supplementary Table S1: Description language for sensory evaluation of Ganmianpi. Supplementary Figure S1: Rarefaction curves and Shannon–Wiener curves of bacterial and fungal communities in slurry.
