# Supplementary material for: Microbial Community Dynamics and Metabolite Changes during Wheat Starch Slurry Fermentation
Source: Foods. 2024 Aug 18;13(16):2586. doi: 10.3390/foods13162586 (PMC11353887; doi:10.3390/foods13162586)
Supplement: Supplementary file 1 [file foods-13-02586-s001.zip › Supplementary Figure.pdf]

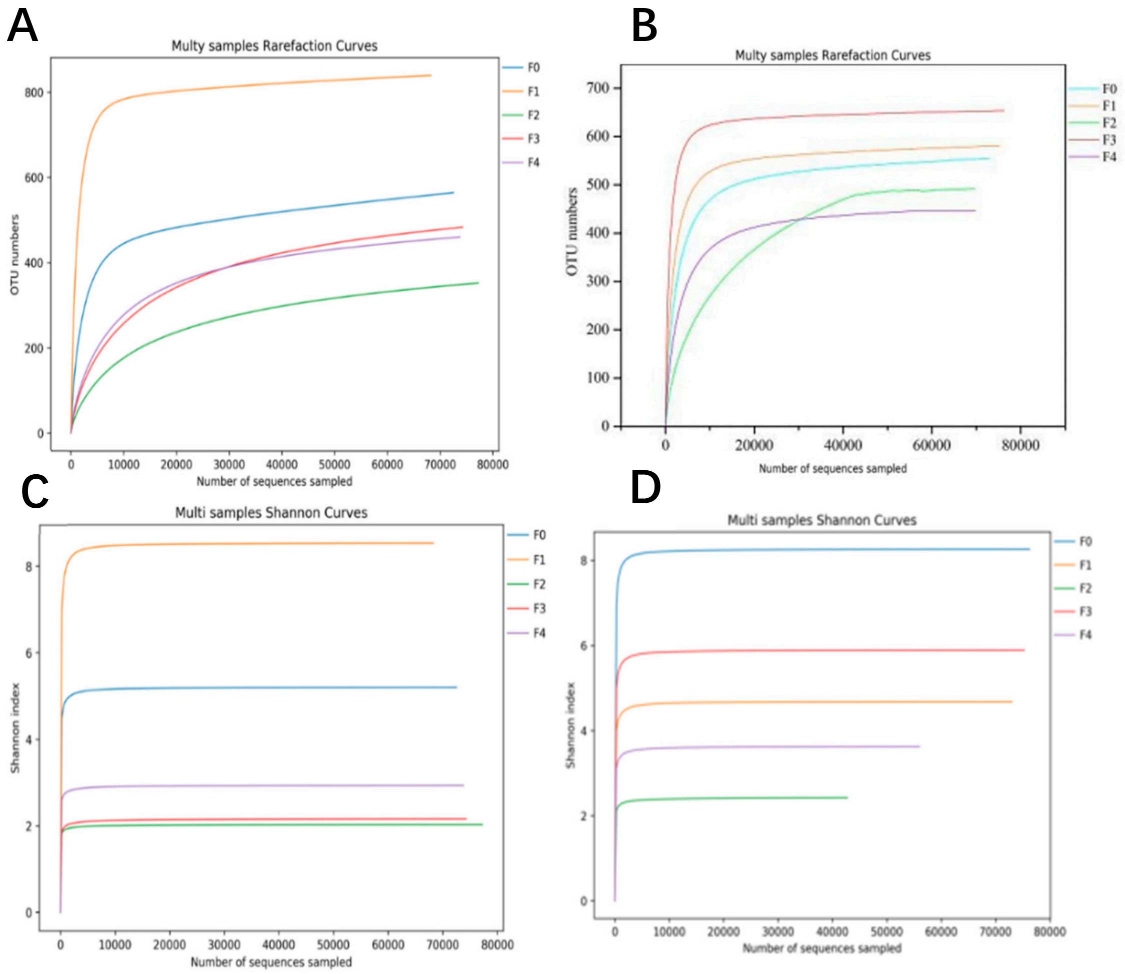


**Supplementary Figure S1**. Rarefaction curves and Shannon-wiener curves of bacterial (A, C) and fungal (B, D) communities in slurry
