# Supplementary material for: Microbial Community Dynamics and Metabolite Changes during Wheat Starch Slurry Fermentation
Source: Foods. 2024 Aug 18;13(16):2586. doi: 10.3390/foods13162586 (PMC11353887; doi:10.3390/foods13162586)
Supplement: Supplementary file 1 [file foods-13-02586-s001.zip › Supplementary Table.pdf]

Supplementary Table S1. Description language for sensory evaluation of Ganmianpi.

Terms	Definition	Evaluation score
Color (10 points)	a. Beige white or light yellow, bright and uniform, translucent	7-10
	b. Beige light yellow, bright but uneven, translucent	4-6
	c. Dull, lustrous and opaque	1-3
Smoothness (10 points)	a. Moist, full, smooth and uniform	7-10
	b. Slightly moist, smooth and uniform	4-6
	c. Dry and uneven	1-3
Elasticity (25 points)	a. Good, tight, and no fragments	19-25
	b. Normal, little fragments	9-18
	c. Bad, incomplete, and lots of fragments	1-8
Toughness (15 points)	a. Easy to press and recover	11-15
	b. Can be pressed, can recover	6-10
	c. Hard to press, and recover	1-5
Chewiness (25 points)	a. Chewy, not easy to break	19-25
	b. Tough, laborious, not easy to break	9-18
	c. Tender, has broken slag	1-8
Flavor (10 points)	a. Fragrant, slightly sour, with the unique flavor of Ganmianpi, long aftertaste	7-10
	b. Fragrant, medium sour taste, has aftertaste	4-6
	c. No fragrance, strong sour taste, poor aftertaste	1-3
